# Compound non-starch polysaccharide enzymes improve growth performance, slaughter performance, immune function, and apparent utilization rate of nutrients in broiler chickens fed a low-metabolizable energy diet

**DOI:** 10.3389/fvets.2023.1162811

**Published:** 2023-05-25

**Authors:** Xing Chen, Guang-min Zhang, Wei-wei Wang, Guo-hua Liu, Hui-yi Cai, Adanan Purba, Ai-juan Zheng

**Affiliations:** Key Laboratory for Feed Biotechnology of the Ministry of Agriculture and Rural Affairs, Institute of Feed Research, Chinese Academy of Agricultural Sciences, Beijing, China

**Keywords:** non-starch polysaccharide enzyme, growth performance, immune function, broiler chickens, slaughter performance, nutrient apparent utilization

## Abstract

This study aimed to investigate the effects of compound non-starch polysaccharide (NSP) enzymes on growth performance, slaughter performance, immune function, and apparent utilization of nutrients in broiler chickens fed a low-metabolizable energy diet. A total of 240 healthy 1-day-old AA broilers (Arbor Acres, 47.2 ± 0.31 g) were randomly divided into four treatment groups, each with six replicate groups and 10 broilers per replicate. The control group was fed a basal diet; the EL-H group was fed the basal diet supplemented with 200 mg/kg compound NSP enzyme, including β-mannanase 5,000 IU/g, β-glucanase 2000 IU/g, xylanase 10,000 IU/g, and cellulase 500 IU/g. The EL-M group was fed the basal diet with 50 kcal/kg metabolizable energy removed, supplemented with 200 mg/kg compound NSP enzyme. Finally, the EL-L group was fed the basal diet with 100 kcal/kg metabolizable energy removed, supplemented with 200 mg/kg compound NSP enzyme. The results showed that feeding with a low-metabolizable energy diet supplemented with compound NSP enzymes did not significantly affect the growth performance of broilers (*p* > 0.05). Compared with the control group, the abdominal fat rate of broilers in the EL-L group was significantly reduced, and that of broilers in the EL-M group was significantly increased (*p* < 0.05). Apparent utilization of dry matter, crude protein, and energy in the diet was lower in the control group than in the EL-L group, but significantly higher in the control group than in the EL-H group (*p* < 0.05). In addition, apparent utilization of crude fiber was significantly increased in the EL-H, EL-M, and EL-L groups compared with the control group (*p* < 0.05). In conclusion, this experiment showed that the addition of 200 mg/kg compound NSP enzyme enabled maintenance of the normal growth and development of broiler chickens fed a low-metabolizable energy diet (replacing 50–100 kcal/kg metabolizable energy). This study provides a theoretical basis for the application of the compound NSP enzyme in broiler chickens.

## Introduction

A corn–soybean meal diet has become the prevailing feed formula used in the broiler industry (1, 2). However, a major issue is that corn and soybean meal contain substantial amounts of non-starch polysaccharides (NSPs), including arabinoxylan, cellulose, and pectin. These NSPs can increase chyme viscosity in the animal gut, slowing down the longitudinal and lateral movement of chyme in the intestine, which ultimately reduces the contact of nutrients such as protein and starch with endogenous protease and amylase (3–5). Furthermore, this also hinders the contact of amino acids and sugars with the intestinal wall (6). The above factors lead to a reduction in diet nutrient utilization in broiler chickens and harm the economic interests of enterprises (7). In addition, the price of soybean oil is closely related to the supply of and demand for soybean meal (8). Reducing costs and increasing economic benefits are considered to be the two major challenges faced by the broiler chicken industry at present.

Studies have shown that 400–450 kcal/kg of energy is not efficiently digested and utilized when broiler chickens are fed a corn–soybean diet; this may be explained by physical encapsulation of undigested fat, protein, and starch in the cell wall or within the fibrous matrix, which the endogenous enzymes of broiler chickens are unable to break down (9–12). Single enzymes, such as α-galactosidase, xylanase, and β-glucanase, are commonly utilized in production to enhance the growth performance of broilers and improve apparent utilization of the diet (13–15). Nevertheless, the structural complexity and composition of poultry feed render it challenging for a single enzyme to achieve the desired results (16). To address this issue, various combinations of NSP enzymes can be selected for production needs; these combinations can produce positive synergistic effects and accelerate the speed of decomposition of substrates.

Against this background, this study aimed to reduce the metabolizable energy required in the diet of broilers by adjusting the proportions of corn, soybean meal, and soybean oil in the broiler diet and, additionally, to further investigate the effects of application of compound NSP enzymes in broilers fed a low-metabolizable energy diet. We hypothesized that supplementation of a low-metabolizable diet with compound NSP enzymes would enhance the normal production performance of broiler chickens. This study provides new ideas and opportunities for enterprises to decrease their expenses and enhance economic benefits through the application of compound NSP enzymes in low-energy diets.

## Materials and methods

### Experimental design and bird management

The feeding trial was conducted according to the guidelines for animal experiments set out by the National Institute of Animal Health. All procedures involving animals, such as welfare and ethical issues, were approved by the Chinese Academy of Agricultural Sciences (ID: AEC-CAAS-20191106).

The experiment adapted a complete randomized design. A total of 240 healthy 1-day-old AA broilers (Arbor Acres, 47.2 ± 0.31 g) were randomly divided into four treatment groups: a control group (CON), and experimental groups EL-H, EL-M, and EL-L. Each group was housed in six replicate cages with 10 birds per cage. The control group received a basal diet, while the EL-H group was given the basal diet supplemented with 200 mg/kg of a compound NSP enzyme. This enzyme contained β-mannanase (5,000 IU/g), β-glucanase (2000 IU/g), xylanase (10,000 IU/g), and cellulase (500 IU/g) by Beijing Challenge Agriculture Science and Technology Co. Ltd. (Beijing, China). The EL-M group received the same enzyme content, but with 50 kcal/kg of metabolizable energy removed from the basal diet. Finally, the EL-L group was supplemented with 200 mg/kg of the compound NSP enzyme but with 100 kcal/kg of metabolizable energy removed from the basal diet.

Broilers were raised according to the AA broiler management guidelines. The light cycle was set to 16 h of light and 8 h of darkness. Broilers were supplied with water *via* nipple drinking systems were provided with granular feed. In the first 0–3 days, the temperature was controlled at 33°C; the temperature was then gradually reduced to 24°C until the end of the test. Broilers were vaccinated against Marek’s disease on the first day and against Newcastle disease and infectious bronchitis on the seventh day. The relative humidity was maintained at 60–70% for the first week and then at 50–60% for the remainder of the study period. The feeds provided to the CON, EL-H, EL-M, and EL-L groups were composed of the same raw materials, and the proportion of raw materials was adjusted to reduce the metabolizable energy according to condition. Broilers were fed and outcome measures analyzed according to two feeding stages: the starter stage (1–21 days) and the grower stage (22–42 days). The composition of the basal diet and corresponding calculated nutrient levels are shown in Table 1.

**Table 1:** Dietary ingredients and nutrient levels in the basal diet (as-fed basis).

Ingredient	Starter stage (1–21 days) (%)				Grower stage (22–42 days) (%)			
	CON	EL-H	EL-M	EL-L	CON	EL-H	EL-M	EL-L
Corn	57.12	58.25	59.49	60.60	59.47	60.59	61.72	62.85
Soybean oil	2.82	1.89	0.92	0.00	4.67	3.74	2.81	1.88
Soybean meal (46% crude protein)	27.34	27.14	26.93	26.76	18.40	18.20	18.00	17.80
Cottonseed meal	3.00	3.00	3.00	3.00	5.00	5.00	5.00	5.00
DDGS	3.00	3.00	3.00	3.00	5.00	5.00	5.00	5.00
Corn protein powder (70% crude protein)	2.00	2.00	2.00	1.98	3.00	3.00	3.00	3.00
NaCl	0.35	0.35	0.35	0.35	0.35	0.35	0.35	0.35
CaHPO_4_	1.26	1.25	1.24	1.24	0.90	0.90	0.89	0.88
Limestone	1.60	1.60	1.61	1.61	1.72	1.73	1.73	1.74
L-lysine hydrochloride	0.64	0.65	0.65	0.65	0.69	0.69	0.70	0.70
DL-methionine	0.20	0.20	0.19	0.19	0.18	0.18	0.18	0.18
L-threonine	0.17	0.17	0.17	0.17	0.17	0.17	0.17	0.17
Choline chloride	0.20	0.20	0.15	0.15	0.15	0.15	0.15	0.15
Premix^1^	0.30	0.30	0.30	0.30	0.30	0.30	0.30	0.30
Total	100.00	100.00	100.00	100.00	100.00	100.00	100.00	100.00
Nutrient levels^2^								
ME, Mcal/kg	3.00	2.95	2.90	2.85	3.15	3.10	3.05	3.00
Crude protein	21.00	21.00	21.00	21.00	19.00	19.00	19.00	19.00
Calcium, %	0.95	0.95	0.95	0.95	0.90	0.90	0.90	0.90
Phosphorus	0.57	0.57	0.57	0.57	0.48	0.48	0.48	0.48
Apparent total tract digestibility phosphorus	0.38	0.38	0.38	0.38	0.35	0.35	0.35	0.35
Methionine	0.53	0.53	0.53	0.53	0.49	0.49	0.49	0.49
Lysine	1.43	1.43	1.43	1.43	1.29	1.29	1.29	1.29

1 The premix provided the following per kg diet: VA 10,000 IU; VD_3_ 2000 IU; VE 10 IU; VK_3_ 2.5 mg; VB_1_ 1 mg; VB_2_ 6 mg; VB_3_ 10 mg; VB_5_ 40 mg; VB_6_ 3 mg; VB_11_ 0.3 mg; VB_12_ 0.01 mg; biotin 0.12 mg; Cu (as copper sulfate) 8 mg; Fe (as ferrous sulfate) 80 mg; Mn (as manganese sulfate) 60 mg; Zn (as zinc sulfate) 40 mg; Se (as sodium selenite) 0.15 mg; I (as potassium iodide) 0.35 mg.

2 Calculated values.

### Sample collection

During the trial period, test chickens’ growth and health were observed, and feed intake and the number and weight of dead chickens were recorded. Body weight and feed intake on the 21st and 42nd days were determined in order to calculate average daily gain (ADG), average daily feed intake (ADFI), and feed:gain ratio (F/G). The data on ADG and ADFI were ultimately corrected for mortality rates.

On the last day of the experiment, after a 12-h fast, 10 mL blood was sampled from the wing vein of each chicken and transferred into tubes without anticoagulant. After centrifugation at room temperature at 3000 × *g* for 15 min, serum was obtained and stored in a 20°C freezing chamber until analysis.

Two broilers per replicate group, of approximate equal body weight, were selected and killed by neck dislocation at the end of the blood collection period. The weight obtained after fasting for 12 h was considered to be the body weight (BW). Dressed weight (DW) refers to weight after bloodletting and feather removal. Dressing percentage (DP) was calculated as DW/BW. Half-eviscerated weight (HEW) refers to the weight of the carcass minus the trachea, esophagus, crop, intestine, spleen, pancreas, gall bladder, reproductive organs, stomach contents, and keratin. Eviscerated weight (EW) was taken as HEW minus the weight of the heart, liver, muscle stomach, glandular stomach, abdominal fat, head, and foot. After stripping the pectoral muscle, leg muscle, and abdominal fat of broilers, pectoral muscle weight (PMW), leg muscle weight (LMW), and abdominal fat weight (AFW) were weighed and recorded. Pectoral muscle rate (PMR), leg muscle rate (LMR), and abdominal fat rate (AFR) were calculated as PMW/EW, LMW/EW, and AFW/EW, respectively.

On the 39th day of the experiment, two broilers from each replicate group with similar weights were placed in cages for feeding and were used for metabolic experiments. Broilers were fed a feed containing exogenous indicators (0.4% TiO_2_). After the test birds had passed a 3-day pre-experiment, a 4-day formal test was conducted. Excreta were collected from each replicate at 09:00 and 16:00 every day, and the apparent rates of metabolism of dry matter (DM), energy (EN), crude protein (CP), and crude fiber (CF) were measured. The freeze-dried excreta samples were ground, passed through a 40-mesh sieve, and stored in a sealed bag for subsequent analysis. Crude protein content was analyzed using a Dumas nitrogen analyzer (FlashSmart N/protein, ThermoFisher, Waltham, MA, United States). A semi-automatic fiber analyzer (A200i, ANKOM, Macedon, NY, United States) was used to analyze crude fiber content. Calorimeters (C200, IKA, Wilmington, NC, United States) were used to analyze energy. The apparent utilization rate of nutrients was calculated as follows:

Apparent utilization rate of DM in the diet (%): [TiO_2_(Excreta)-TiO_2_(Diet)]/TiO_2_(Excreta) × 100.

Apparent utilization rate of nutrients in the diet (%): [1-TiO_2_(Diet)/TiO_2_(Excreta) × Nutrients(Excreta)/Nutrients(Diet)] × 100.

### Serum biochemistry

Serum total protein (TP), albumin (ALB), globulin (GLB), total cholesterol (TCHO), triglyceride (TG), low-density lipoprotein (LDL), high-density lipoprotein (HDL), blood urea nitrogen (BUN), and uric acid (UA) were determined using a fully automatic biochemical analyzer (AU5800, American Beckman Coulter Co., Ltd., United States).

### Serum immunoglobulin

Serum immunoglobulin G (IgG, H106-1-1), immunoglobulin M (IgM, H109-1-2), and immunoglobulin A (IgA, H108-1-2) were determined using commercial enzyme-linked immunosorbent assay (ELISA) kits (Jiangsu Nanjing Jiancheng Biotechnology Co., Ltd., Jiangsu, China).

### Statistical analyses

All data were analyzed using the general linear model (GLM) in SAS 9.4 (SAS Institute Inc., Cary, NC, United States). The statistical model employed for the study was as follows: Y_ijk_ = μ + T1 + Σ_ijk_, where Y_ijk_ = the overall observation (production), μ = population mean, T1 = effect of compound non-starch polysaccharide enzymes in broiler chickens fed with a low-metabolizable energy diet, Σ_ijk_ = residual effects. Differences among treatments were compared using Duncan’s multiple range tests. The results are presented in form of means with standard error of the mean (SEM). Differences were considered significant at *p* < 0.05.

## Results

### Growth performance

Table 2 illustrates the effects of the compound non-starch polysaccharide enzyme on the growth performance of broilers. The results indicated that there was no significant difference in ADG, ADFI, or F/G between the CON group and the EL-H, EL-M, or EL-L groups during any time period (days 1–21, days 22–42, or days 1–42; *p* > 0.05).

**Table 2:** The effects of compound non-starch polysaccharide enzyme on the growth performance of broiler chickens.

Item	CON	EL-H	EL-M	EL-L	SEM	*p*-value
Starter stage (1–21 days)						
ADG, g/d	45.87	46.79	46.34	45.86	0.235	0.468
ADFI, g/d	53.35	54.40	53.64	54.03	0.360	0.772
F/G	1.16	1.16	1.16	1.18	0.005	0.477
Grower stage (22–42 days)						
ADG, g/d	92.83	95.62	93.81	96.87	1.120	0.610
ADFI, g/d	157.08	155.07	154.73	153.91	1.458	0.901
F/G	1.70	1.62	1.65	1.59	0.018	0.153
Overall (1–42 days)						
ADG, g/d	66.93	68.20	67.41	68.13	0.464	0.757
ADFI, g/d	101.60	100.79	100.72	99.68	0.704	0.836
F/G	1.52	1.48	1.49	1.46	0.009	0.105

ADG, average daily gain; ADFI, average daily feed intake; F/G, feed/gain. CON group, control group fed a basal diet; EL-H group, basal diet +200 mg/kg compound NSP enzyme; EL-M group, removal of 50 kcal/kg metabolizable energy from the basal diet +200 mg/kg compound NSP enzyme; EL-L group, removal of 100 kcal/kg metabolizable energy from the basal diet +200 mg/kg compound NSP enzyme.

### Slaughter performance

The effects of the compound non-starch polysaccharide enzyme on the slaughter performance of broilers are shown in Table 3. The results indicated that there was no significant difference in DP, HEWR, EWR, PMR, or LMR among the groups (*p* > 0.05). However, broilers in the EL-H group had the highest AFR (*p* < 0.05), while the AFR of broilers in the EL-L group was significantly lower compared with the other groups (*p* < 0.05).

**Table 3:** The effects of compound non-starch polysaccharide enzyme on the slaughter performance of broiler chickens.

Item	CON	EL-H	EL-M	EL-L	SEM	*p*-value
SR, %	92.01	90.21	91.32	92.04	0.232	0.725
HEW, %	87.00	87.67	86.26	87.08	0.331	0.413
EW, %	77.10	73.89	75.44	77.16	0.323	0.825
PMR, %	49.06	51.68	48.84	48.66	0.218	0.621
LMR, %	32.40	33.46	33.58	35.18	0.167	0.142
AFR, %	1.36^b^	1.42^ab^	1.52^a^	1.17^c^	0.051	0.032

SR, slaughter rate; HEW, half-eviscerated weight; EW, eviscerated weight; PMR, pectoral muscle rate; LMR, leg muscle rate; AFR, abdominal fat rate. CON group, control group fed a basal diet; EL-H group, basal diet +200 mg/kg compound NSP enzyme; EL-M group, removal of 50 kcal/kg metabolizable energy from the basal diet +200 mg/kg compound NSP enzyme; EL-L group, removal of 100 kcal/kg metabolizable energy from the basal diet +200 mg/kg compound NSP enzyme. Different lowercase letters alongside data in the same row indicate significant differences (*p* < 0.05).

### Serum biochemistry

Table 4 shows the effects of the compound non-starch polysaccharide enzyme on the serum biochemistry of broilers. Compared with the CON group, there was no significant difference in TP, ALB, GLB, TCHO, TG, LDL, HDL, BUN, or UA of broiler serum for the EL-H, EL-M, or EL-L group (*p* > 0.05).

**Table 4:** The effects of compound non-starch polysaccharide enzyme on the serum biochemistry of broiler chickens.

Item	CON	EL-H	EL-M	EL-L	SEM	*p*-value
TP, mg/mL	17.42	16.39	19.13	20.36	0.930	0.481
ALB, mg/mL	7.4	7.07	7.60	8.17	0.285	0.627
GLB, g/L	10.02	9.32	11.52	12.19	0.681	0.457
TCHO, μmol/dL	1.50	1.35	1.76	1.64	0.074	0.237
TG, mg/mL	0.28	0.26	0.34	0.38	0.021	0.141
LDL, ng/mL	0.44	0.43	0.39	0.78	0.061	0.058
HDL, ng/mL	1.54	1.37	1.47	1.74	0.065	0.250
BUN, mg/mL	0.17	0.28	0.17	0.17	0.021	0.937
UA, μmol/L	155.04	147.41	149.48	196.74	12.408	0.492

TP, total protein; ALB, albumin; GLB, globulin; TCHO, total cholesterol; TG, triglyceride; LDL, low-density lipoprotein; HDL, high-density lipoprotein; BUN, blood urea nitrogen; UA, uric acid. CON group, control group fed a basal diet; EL-H group, basal diet +200 mg/kg compound NSP enzyme; EL-M group, removal of 50 kcal/kg metabolizable energy from the basal diet +200 mg/kg compound NSP enzyme; EL-L group, removal of 100 kcal/kg metabolizable energy from the basal diet +200 mg/kg compound NSP enzyme. Different lowercase letters alongside data in the same row indicate significant differences (*p* < 0.05).

### Serum immunoglobulin

The effects of the compound non-starch polysaccharide enzyme on serum immunoglobulin in broilers are shown in Table 5. The serum IgA concentration of broilers in the EL-L group was significantly lower than that of broilers in the other groups (*p* < 0.05). However, there was no significant difference in IgA content between the CON, EL-H, and EL-M groups (*p* > 0.05). Serum IgM and IgG levels did not differ significantly among the CON, EL-H, EL-M, and EL-L groups (*p* > 0.05).

**Table 5:** The effect of compound non-starch polysaccharide enzyme on the immunoglobulin of broiler chickens.

Item	CON	EL-H	EL-M	EL-L	SEM	*p*-value
IgA, μg/mL	2.23^a^	2.24^a^	2.09^a^	1.27^b^	0.104	< 0.001
IgM, μg/mL	1.46	1.54	1.62	1.63	0.039	0.393
IgG, μg/mL	3.86	4.13	4.04	4.18	0.064	0.314

IgA, immunoglobulin A; IgM, immunoglobulin M; IgG, immunoglobulin G. CON group, basal diet in the control group; EL-H group, basic diet +200 mg/kg compound NSP enzyme; EL-M group, removal of 50 kcal/kg metabolizable energy +200 mg/kg compound NSP enzyme from the basic diet; EL-L group, removal of 100 kcal/kg metabolizable energy +200 mg/kg compound NSP enzyme from the basic diet. Different lowercase letters in the shoulders of the data in the same row indicate significant differences (*p* < 0.05).

### Apparent utilization rate of nutrients

The effects of compound non-starch polysaccharide enzyme on the apparent utilization rate of nutrients in the broilers’ diets are shown in Table 6. Compared with the CON group, the EL-H group showed significant reductions in utilization of DE, CP, and EN (*p* < 0.05). In contrast, the EL-L group exhibited a significant improvement in apparent utilization rates for DE, CP, and EN compared to the other groups (*p* < 0.05). Furthermore, supplementation of the diet with compound NSP enzyme (in the EL-H, EL-M, and EL-L groups) significantly enhanced the apparent utilization rate of *CF* in broilers, in comparison to the CON group. However, no significant differences were observed among the EL-H, EL-M, and EL-L groups (*p* > 0.05).

**Table 6:** The effects of compound non-starch polysaccharide enzyme on the apparent utilization rate of nutrients in the feed of broiler chickens.

Item	CON	EL-H	EL-M	EL-L	SEM	*p*-value
DM, %	73.06^b^	68.80^c^	74.93^b^	77.37^a^	0.979	< 0.001
CP, %	58.43^b^	55.36^c^	60.54^b^	65.67^a^	1.194	< 0.001
EN, %	76.65^b^	74.03^c^	76.39^b^	79.96^a^	0.682	< 0.001
*CF*, %	13.37^b^	26.43^a^	29.28^a^	27.77^a^	1.983	< 0.001

DM, dry matter; CP, crude protein; EN, energy; CF, crude fiber. CON group, control group fed a basal diet; EL-H group, basal diet +200 mg/kg compound NSP enzyme; EL-M group, removal of 50 kcal/kg metabolizable energy from the basal diet +200 mg/kg compound NSP enzyme; EL-L group, removal of 100 kcal/kg metabolizable energy from the basal diet +200 mg/kg compound NSP enzyme. Different lowercase letters alongside data in the same row indicate significant differences (*p* < 0.05).

## Discussion

The corn–soybean meal diet is rich in NSPs, including arabinoxylan, β-glucan, cellulose, and pectin (17, 18). These NSPs can increase the viscosity of intestinal digest, decrease the diffusion rate of the substrate and digestive enzymes, and ultimately reduce the digestion and absorption of nutrients, leading to poor growth performance in broilers (19). Additionally, β-mannan is a type of hemicellulose, occurring in the form of glucomannan and galactomannan, which negatively affects body weight gain (BWG), feed intake (FI), and feed conversion rate (FCR) in broilers (20, 21). Previous studies have demonstrated that a diet supplemented with 500 mg/kg or 1.7 × 10^8^ U/kg β-mannanase significantly enhances BWG in broilers (9, 22). Furthermore, adding β-mannanase to a low-energy diet does not affect the growth performance of broilers, but it does increase the apparent rates of metabolism of DM and *CF* in the diet (23). These findings suggest that β-mannanase can improve utilization of feed nutrients by reducing intestinal chyme viscosity and increasing carbohydrate utilization (24, 25). Research has shown that β-glucanase effectively decreases levels of non-starch polysaccharides (NSPs) and crude fiber in wheat or barley diets (26–28). Similarly, the beneficial effects of xylanase on the growth performance of broilers have been widely reported (29, 30). In particular, supplementation of xylanase significantly enhances broiler performance and improves feed utilization (31, 32). Cellulase, as a complex enzyme system, binds specifically to cellulose substrates and decomposes them into glucose through the synergistic action of multiple components (33, 34). A previous study has shown that use of a compound NSP enzyme significantly enhances growth performance and feed nutrient utilization in broilers (35, 36). The selection and collocation of complex NSP enzymes should be designed according to different feed formulations. The content and types of NSPs found in formulas composed of different feed ingredients are different, so it is necessary to use different doses and types of NSP enzymes for degradation. In this experiment, the diet was composed of corn (57–62%), soybean meal (27–17%), and a small amount of cottonseed meal (3%), which results in sensitivity among the broiler chickens to the intake of arabinoxylan, β-glucan, and cellulose. Therefore, supplementation of compound NSP enzymes (β-mannanase, β-glucanase, xylanase, and cellulase) in the diet can solve the negative effects produced by excessive NSPs and improve the digestion and utilization rate of nutrients. This may explain why feeding with a low-metabolizable energy diet supplemented with an NSP enzyme did not affect the production performance of broilers. Furthermore, the apparent utilization rate of DM, CP, EN, and *CF* appeared to increase significantly in the case of a low-metabolizable energy diet with the addition of 200 mg/kg compound NSP enzymes. This result indicates that the compound NSP enzyme effectively enhanced utilization of nutrients in the feedstuff. Therefore, the compound NSP enzyme benefits broilers fed a low-metabolizable energy diet. It reduces the viscosity of intestinal chyme, increasing nutrient absorption in the intestine. Additionally, it improves utilization of raw feedstuff, ultimately resulting in maintenance of normal growth and development in broilers.

Slaughter performance is the main index used to evaluate the performance of livestock products (37). Previous studies have indicated that the use of this compound enzyme does not significantly affect broilers’ slaughter performance (23, 38, 39). Our study found that the EL-M group had the highest abdominal fat rate. This suggests that supplementing the diet of broilers with 200 mg/kg NSP enzyme can lead to increased abdominal fat deposition, even with a 100-kcal/kg reduction in metabolizable energy in the feed. However, we also observed that the abdominal fat rate of broilers in the EL-L group was significantly lower than that of broilers in the other groups. This difference could be attributed to the experimental design and the type of complex NSP enzyme used. Broilers fed a low-metabolizable energy diet require less energy to sustain their growth needs, which decreases the energy used for abdominal fat deposition (40). This suggests that broilers prioritize the use of nutrients obtained from a low-metabolizable energy diet in the maintenance of growth requirements rather than in storage.

Previous studies have indicated a strong correlation between dietary metabolizable energy and the immune function of broilers. In particular, a high-metabolizable energy diet has been reported to increase immunoglobulin content in the plasma of broilers (41, 42). In this study, we aimed to design a low-metabolizable energy diet by reducing soybean oil content. Soybean oil contains linoleic acid, an n-6 polyunsaturated fatty acid (PUFA), which has been found to play a regulatory role in the immune function of broilers (43, 44). In this experiment, we observed a significant decrease in serum IgA levels in broilers in the EL-L group. Serum IgA plays a crucial role in mucosal immune response, which is an essential indicator of immune function in broilers (45, 46). Previous studies have demonstrated that malnutrition reduces body fat content, affecting the immune signaling function of adipocytokines and the body’s overall immune capacity (47). In this study, the EL-L group sustained the levels of growth and development typical of broilers and exhibited a significant decrease in abdominal fat content. We speculate that the reduced quantity of soybean oil in the diet may have contributed to the decrease in body fat content, which could potentially affect the immune function of the broilers. However, further research is necessary to determine the specific mechanism behind this potential reduction in immune function.

## Conclusion

In this experiment, the supplementation of a low-metabolizable diet with 200 mg/kg of NSP complex enzymes (β-mannanase, β-glucanase, xylanase, and cellulase) significantly improved the growth and development of broiler chickens. The application of the compound NSP enzyme significantly reduced the cost of feed (e.g., due to reduction in the required soybean oil content), and also provided high economic value. Based on this study’s results, we recommend adding 200 mg/kg of compound NSP enzymes to the diet as a replacement for 50–100 kcal/kg of metabolizable energy.

## Data availability statement

The raw data supporting the conclusions of this article will be made available by the authors, without undue reservation.

## Ethics statement

The feeding trial was conducted according to the guidelines for animal experiments set out by the National Institute of Animal Health. All procedures involving animals such as welfare and ethical issues were approved by the Chinese Academy of Agricultural Sciences (ID: AEC-CAAS-20191106).

## Author contributions

G-mZ acquired the funding. XC and G-mZ conceptualized and designed the study. XC and W-wW conducted the animal experiments and the chemical analysis and analyzed the data. XC wrote the original draft of the manuscript. G-hL, H-yC, AP, and A-jZ reviewed and revised the draft. All the authors agreed to publication of the manuscript.

## Funding

This work was supported by the China Agricultural Research System (CARS-41).

## Conflict of interest

The authors declare that the research was conducted in the absence of any commercial or financial relationships that could be construed as a potential conflict of interest.

## Publisher’s note

All claims expressed in this article are solely those of the authors and do not necessarily represent those of their affiliated organizations, or those of the publisher, the editors and the reviewers. Any product that may be evaluated in this article, or claim that may be made by its manufacturer, is not guaranteed or endorsed by the publisher.
